# High Follicle-Stimulating Hormone Level Associated With Risk of Rheumatoid Arthritis and Disease Activity

**DOI:** 10.3389/fendo.2022.862849

**Published:** 2022-04-22

**Authors:** Xianhui Zhang, Pengyan Qiao, Qianyu Guo, Zixie Liang, Jie Pan, Fengping Wu, Xuexue Wang, Liyun Zhang

**Affiliations:** ^1^Department of Rheumatology, Third Hospital of Shanxi Medical University, Shanxi Bethune Hospital, Shanxi Academy of Medical Sciences, Tongji Shanxi Hospital, Tongji Medical College, Huazhong University of Science and Technology, Taiyuan, China; ^2^Department of Biomedical Engineering, Duke University Pratt School of Engineering, Durham, NC, United States; ^3^Department of Pathology, Stanford University School of Medicine, Palo Alto, CA, United States; ^4^School of Basic Medical Sciences, Shanxi Medical University, Taiyuan, China; ^5^Department of Rheumatology, Shanxi Bethune Hospital, Shanxi Academy of Medical Sciences; Tongji Shanxi Hospital, Tongji Medical College, Huazhong University of Science and Technology, Taiyuan, China

**Keywords:** FSH, menopause, rheumatoid arthritis, disease activity, sex hormones

## Abstract

**Background:**

The prevalence of rheumatoid arthritis (RA) has significant gender and age difference. The peak age of RA is consistent with the age of menopause, which is accompanied by a sharp increase in serum follicle-stimulating hormone (FSH) level. This study aims to identify the FSH levels in female RA patients and the relationship with diseases activity.

**Methods:**

In total, 79 female RA patients and 50 age-matched controls were included in our study. Serum sex hormones levels were measured using chemiluminescence. RA patients were grouped by FSH quartile. Disease activity and inflammatory marks were analyzed among groups.

**Results:**

Lower sex hormones and higher gonadotropin were found in RA patients. Serum FSH level was significantly higher in RA patients than in the age-match controls (57.58 ± 15.94 vs. 43.11 ± 19.46, p=0.025). Even after adjusting for age (OR: 1.071; 95%CI: 1.006-1.139; p = 0.031), luteinizing hormone (LH), estradiol (E), and testosterone (T) OR: 1.066; 95%CI: 1.003-1.133; p = 0.039), the OR were still more than one. RA patients in the higher quartiles had higher ESR, DAS28-ESR and DAS28-CRP (p<0.05) than the lowest quartile. Besides, menopause age was significantly related with onset age in post-menopause RA patients (r = 0.432, p =0.008).

**Conclusion:**

High FSH appears to be a risk factor for RA and is positively associated with their disease activity. Early menopause might be an essential factor of RA.

## Introduction

Rheumatoid arthritis (RA) is a chronic autoimmune disease characterized by synovitis, pannus formation, and the destruction of bone and cartilage (1). There are obvious gender differences in the prevalence of most autoimmune diseases, whereby females are generally more susceptible than males. For RA, the incidence in females and males was approximately 2-4:1 (2). The average age at diagnosis of RA patients is 40-60 years, which is consistent with the age of menopause. Early menopause has been reported as an independent risk factor for RA (3). Menopause-related sex hormones are believed to play an essential role in the pathogenesis of RA.

The decline in estrogen is the most prominent feature of menopause and has been considered the leading cause of menopausal complications in the past few decades (4, 5). Estrogen has complex pro-inflammatory and anti-inflammatory roles, which vary by the doses or receptor subtypes. Low levels of estrogens after menopause stimulate pro-inflammatory pathways and enhance proinflammatory cytokine production, whereas high levels can boost Th-2 anti-inflammatory pathways and humoral immunity (6). However, the effect of exogenous estrogen on RA has been reported with controversial results. Early studies have shown that exogenous estrogen has a protective impact on RA (7, 8). These findings were questioned by subsequent studies (9, 10). A meta-analysis of 12 studies reveals that oral contraceptives have no protective effect on the risk of RA in women (11). In addition, postmenopausal hormone therapy (PTH) has not been proved to improve joint pain scores, swelling and prevent new joint pains in RA patients. Besides, antihormonal treatment for breast cancer patients does not seem to increase the risk of RA (12). These findings indicated that factors other than estrogen could also cause autoimmune disorders in RA patients after menopause.

Due to the negative feedback of the gonadal axis, the levels of FSH also increase during menopause transition (4). High FSH levels have been considered to be essential in menopausal osteoporosis, obesity, and hepatic gluconeogenesis (13). Besides, the influence of FSH on perimenopausal immune factors has also attracted attention. Some studies have found that FSH could enhance the expression of the pro-inflammatory cytokines interleukin 1 (IL-1 β), IL-6, and tumor necrosis factor α (14, 15). In this study, we investigated FSH levels in women with RA and evaluated the association between FSH and RA disease activity.

## Material and Methods

### Study Design and Participants

Seventy-nine female patients diagnosed with RA thirty and 50 age-matched healthy control (HC) were included in the study. This study is a prospective analysis. All patients were hospitalized in the Department of Rheumatology, Shanxi Bethune Hospital, Third Hospital of Shanxi Medical University, and met the 1986 American College of Rheumatology classification criteria. The healthy controls were obtained from the physical examination center of Children’s Hospital of Shanxi and Women Health Center of Shanxi. Participants with premature ovarian failure, bilateral ovariectomy, hormone replacement therapy, contraceptives, corticosteroids > 7.5 mg daily, or malignant tumors were excluded. The controls had no sign of arthritis or other inflammation. The study was approved by the Ethics Committee of Shanxi Bethune Hospital. Informed consent of each participant has been obtained.

### Markers of Disease Activity

All data were collected from questionnaires and medical records, including age, onset age, menopause age, body mass index (BMI), patient global assessment (PGA), tender joint count (TJC), swollen joint count (SJC), rheumatoid factor (RF), erythrocyte sedimentation rate (ESR) and C-reactive protein (CRP). The 28-joint disease activity score ESR/CRP (DAS28-ESR/DAS28-CRP) was used to assess the disease activity in RA patients, calculated with PGA, TJC, SJC, ESR, and CRP.

### Laboratory Testing

The blood of perimenopausal and postmenopausal women was collected randomly. Venipuncture was performed on days 3–6 of a regular menstrual cycle for premenopausal women to exclude cyclical differences in sex hormones. All blood samples were collected after overnight fasting and stored at −80°C immediately. Chemiluminescence (Cobas E601, Roche, Switzerland) was used for the quantitative determination of sex hormones, including estradiol (E), testosterone (T), progestin (PROG), FSH, luteinizing hormone (LH), prolactin (PRL).

### Statistical Analyses

All data were analyzed through SPSS software for statistics (IBM SPSS 21.0, IBM Corp., Armonk, NY, USA). Continuous variables were expressed with mean and standard deviation (SD) or median and interquartile according to the normal or nonnormal distribution. Categorical variables are expressed as numbers and percentages; The Student’s t-test or the Mann–Whitney U-test was used to compare the differences between RA patients and healthy controls. A logistic regression model was used to evaluate the FSH in RA and controls with adjustments for age, E2 and T. One-way analysis of variance (ANOVA) was used to compare the clinical parameters among FSH quartiles. A Spearman rank correlation analysis was used to assess the relationship between onset age and menopause age in RA patients. All statistical tests are two-tailed, and p <0.05 is considered statistically significant.

## Results

### Sex Hormone Levels in RA Patients and Healthy Controls

Seventy-nine females with RA and 50 age-matched healthy women were included in the study. The sex hormone levels are shown in **Table 1**. There was no significant difference in BMI between groups. There was also no significant difference in E2 and PROG between patients and controls (p>0.2). However, compared with controls, there was a trend for GnRH, including FSH, LH and PRL, to be higher in patients, despite the difference in LH and PRL being insignificant. The level of FSH was significantly increased in RA patients. Besides, the T level in RA patients was significantly lower than in the control group.

**Table 1:** Sex hormone levels in RA patients and healthy controls.

	RA patients (n=79)	Healthy control (n=50)	P-value
Age (years)	57.07 ± 4.763	55.69 ± 4.127	0.505
BMI (kg/m2)	22.92 ± 2.93	23.59 ± 3.15	0.475
Menopause n (%)	46(58.23)	28(54)	0.637
E2 (pmol/L)	18.35(18.35-39.10)	24.98(18.35-31.63)	0.323
T (nmol/L)	0.11(0.09-0.53)	0.61(0.28-0.67)	0.028*
PROG (ng/ml)	0.15 ± 0.12	0.24 ± 0.46	0.235
FSH (mIU/ml)	57.58 ± 15.94	43.11 ± 19.46	0.025*
LH (mIU/ml)	35.25 ± 15.16	25.08 ± 14.69	0.128
PRL (Uiu/ml)	329.0(277.43-420.15)	237.85(196.78-344.30)	0.143

All data are expressed as mean ± SD or median (interquartile range). *p < 0.05.

### FSH Is an Independent Risk Factor for RA Patients

A logistic regression analysis was conducted to assess whether high FSH affects RA. As shown in **Table 2**, the unadjusted OR was 1.053. The OR was still higher after adjusting for age, E2 and T.

**Table 2:** Logistics regression analysis of FSH levels in RA patients. *p < 0.05.

	B	SE	OR (95%CI)	P-value
univariable model	0.052	0.023	1.053 (1.006-1.103)	0.027
multivariable model 1*	0.068	0.032	1.071 (1.006-1.139)	0.031
multivariable model 2**	0.062	0.028	1.066 (1.003-1.133)	0.039

Unstandardized coefficients (B), corresponding standard error (SE), odds ratio (OR), 95% confidence interval (CI).

*Adjusted for age, **adjusted for E2 and T.

### Markers of Disease Activity in RA Patients With Different FSH Levels

**Figure 1:**
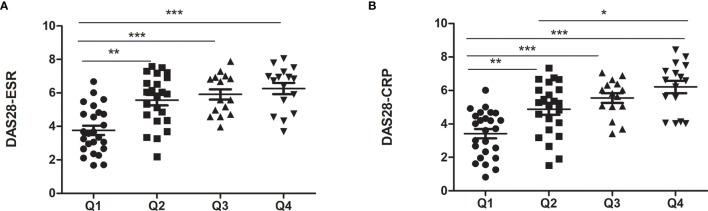
Markers of disease activity in RA patients with different FSH levels. **(A)** for DAS28-ESR; **(B)** for DAS28-CRP. *p < 0.05, **p < 0.01, ***p < 0.001.

We divided RA patients into four groups based on the quartiles of FSH level. As shown in **Table 3**, the quartile ranges of FSH in RA patients were <42.88 mIU/ml (Q1), 42.88-52.97 mIU/ml (Q2), 52.97-67.78 mIU/ml (Q3) and >67.78 mIU/ml. There was no significant difference in age and E2 among the four groups. On the other hand, LH increased with FSH quartiles (p=0.037). As the FSH quartile increased, the mean values of ESR and CRP levels increased from 35.33 to 55 mg/L and 14.21 to 31.85, respectively. Similarly, DAS28-CRP and DAS28-ESR were significantly higher in the upper quartile than in the lowest quartile (**Figure 1**). However, there were no significant differences in markers of disease activity among quartiles 2, 3 and 4.

**Table 3:** Clinical characteristics and sex hormone levels in RA patients with different FSH levels.

	Quartil 1	Quartil 2	Quartil 3	Quartil 4	P-value
N	25	23	15	16	–
FSH levels	~42.88	42.88-52.97	52.97-67.78	67.78~	–
Age (years)	55.33 ± 8.39	59.00 ± 5.35	61.00 ± 5.35	61.50 ± 4.51	0.430
Menopause n (%)	11(44)	15(65.22)	8(53.33)	12(75)	0.209
RF (IU/mL)	54.04 ± 47.89	89.36 ± 56.90	161.02 ± 154.44	92.58 ± 105.59	0.289
E2 (pmol/L)	34.05 ± 13.60	37.46 ± 23.47	19.47 ± 2.24	24.26(18.35-52.35)	0.503
LH (mIU/ml)	23.6 ± 6.38	28.13 ± 10.38	33.11 ± 6.77	54.93 ± 16.05	0.037*
ESR (mg/L)	35.33 ± 32.33	50 ± 26.96	49.75 ± 22.54	55 ± 12.91	0.04*
CRP (mm/h)	14.21 ± 16.66	28.62 ± 14.49	27.73 ± 23.51	31.85 ± 41.87	0.061

*p < 0.05.

### Association Between Menopause Age and Onset Age in RA Patients

**Figure 2:**
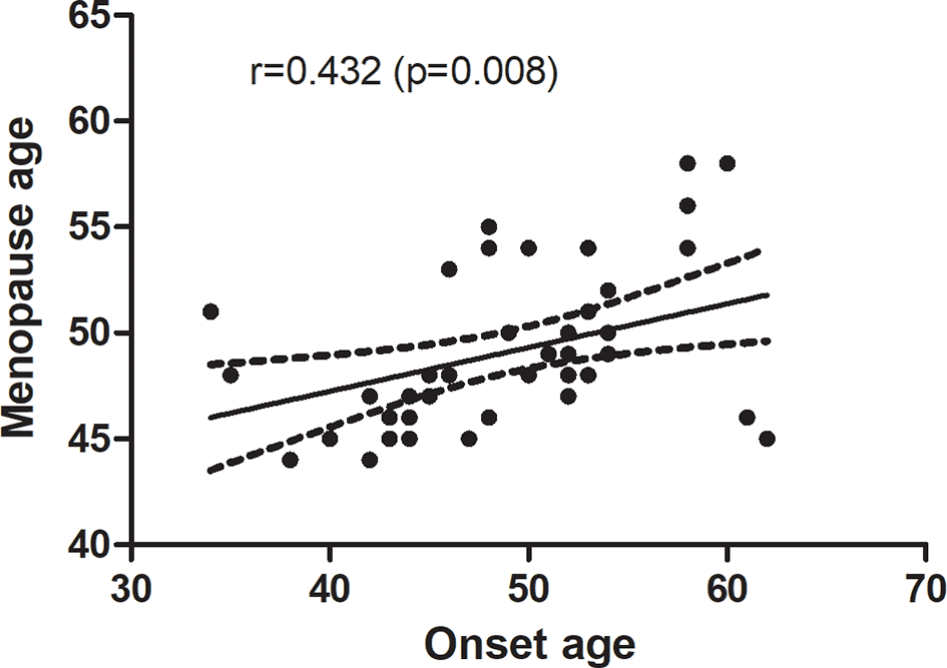
Correlation between menopause age with the onset age of RA patients.

There were 46 post-menopause RA patients included in the study. To address whether menopause age correlates with onset age in RA patients, we performed a correlation analysis, and menopause age was significantly positively correlated with the age of onset of RA patients (r = 0.432, p =0.008) (**Figure 2**).

## Discussion

Although the gender and age bias in autoimmune disease (AID) has long been documented, the mechanisms by which it occurs remain unclear. Our previous study surveyed 379 RA patients and found the female to male RA patient ratio was 2.83, and the peak age of RA females was 51-60 years (data not shown). The peak incidence of RA in women was also consistent with menopausal years in other studies (16). Autoimmune diseases are closely related to aging. They share a cassette of similar clinical symptoms, such as hair loss, osteoporosis, etc. However, this field has not caused enough attention. Autoimmune diseases and aging may share a common signal transduction pathway and mechanism of chronic inflammation. TNF-α is a classic pro-inflammatory factor, which is not only increased in most autoimmune diseases, but also significantly increased in the elderly and related degenerative diseases (17).

Menopause is accompanied by increased GnRH and decreased sex hormone due to ovarian failure. Age is an independent risk of menopause, so age-matched healthy women are selected as controls. In this study, we found that compared with the control group, female patients with RA tend to have higher GnRH and lower sex hormones, which indicates that the prevalence of ovarian failure in RA patients is high. Recently, we conducted a meta-analysis of the assessment of ovarian failure by serum anti-müllerian hormone in RA patients and obtained similar results (18). For GnRH, the increase in LH and PRL did not reach statistical significance. However, FSH was significantly increased in RA patients compared to healthy controls. A case-control study of men also found that higher FSH is related to RF-negative patients (19). However, other studies have not shown a significant difference in FSH between patients and controls (20, 21). For sex hormone, there were no significant differences in E2 and PROG between the two groups, whereas T decreased obviously in RA patients. This significant decrease was due to low E2 and PROG levels in the two groups. Androgens are considered to be immunosuppressive. And lower testosterone was also found in male RA (22, 23). Androgen deprivation therapy for prostate cancer patients was reported to have an increased risk of developing RA (24, 25). Similar to the findings in men, lower testosterone was observed to be associated with female RA patients in our study. However, contrary to previous results in men, higher levels of T were observed in postmenopausal RA patients (26). This inconsistency may be due to the heterogeneity of the disease and the enrolled patients. Larger sample sizes and different phenotypes are needed further to investigate the changes in sex hormones in RA.

We performed a multivariate logistic regression analysis to determine whether elevated FSH levels are independently associated with RA. The results showed that high circulating FSH levels were an independent risk factor for RA. After adjusting potential confounders, the risk remains. Mitra Pikwer also reported that high FSH was associated with the risk of RF-negative RA men after adjusting smoking and BMI [OR: 11.5 (1.46 to 91.1)] (19). Although they found that lower FSH was associated with the risk of RF-positive RA men, the average age of RF-negative RA was markedly higher than that of RF-positive in their study. The age of RF-negative RA is similar to patients in our study. These results indicated that high FSH might affect RA in older adults of both sexes. There is growing evidence that FSH is associated with increased risk of osteoporosis, metabolic factors, renal dysfunction in peri- and post-menopausal women (27–29). The prevalence of osteoporosis and metabolic dysfunction is also higher in RA patients. A recent study published in Nature showed brain is also a target for FSH, and FSH blockade improves cognition in mice with Alzheimer’s disease (30). High FSH may mediate these interactions.

Studies have shown that hormonal status affects the disease characteristics of women with RA. In addition to changes in hormone levels, menopause also leads to increased production of cytokines, such as IL-1, IL-6, and tumor necrosis factor (TNF) (31), which are also grown in RA patients. However, few researches have investigated the relationship between serum FSH levels and inflammatory status in perimenopausal RA women. To investigate whether FSH levels are related to the disease activity of RA, we stratified patients by FSH quartiles. Compared with other quartiles, markers of disease activity in the lowest quartile were significantly lower. A cross-sectional study found that instead of LH level, FSH levels are considerably associated with DAS28 by investigating the effects of Methotrexate (MTX) on gonadotropins in RA patients (32). AS Kass et al. further found a significant correlation between LH and FSH and other key cytokines by evaluating the serum LH, FSH levels, and 24 cytokines of 20 RA patients and 19 controls (20). These findings suggested that FSH may play a pro-inflammatory effect in RA. A recent study on apical periodontitis found that FSH aggravated inflammation by enhancing the expression of IL-1 β, IL-6, TNF and Toll-like receptor 4 in human periodontal ligament cells (15). In our follow-up study, FSH receptor expressions were also found in the various immune cell, which may mediate cell proliferation, differentiation and immune function. This may help partly explain why disease activity begins or worsens during postpartum and menopause when FSH increases. Due to the small sample size, no significant differences were observed among quartiles 2, 3 and 4, except DAS28-CRP between Q2 and Q4.

Significant changes were observed in LH in different FSH quartiles because they are similarly regulated and therefore have corresponding physiological changes. RA patients with early age at menopause had higher mean patient global and pain scores and were more likely to be a rheumatoid factor (RF) positive (33). Therefore, we performed a correlation analysis between menopause age and onset age in 46 post-menopause female RA patients and the results showed a significant positive correlation between them. However, their causal relationship needs further study.

Our study also has several limitations. Firstly, the sample size was small. Seventy-nine female RA patients were enrolled in our study, with only nearly 20 patients in each quartile. As a result, no expected trends were found in FSH quartiles 2,3 and 4. Secondly, the impact of RA treatment was not considered. Although in our previous study, DMARDs treatment had no harmful effect on the ovarian function of RA patients (18), their impact on GnRH is still unclear. More factors should be taken into account in further research. Thirdly, male patients were not included, who account for one-third of RA patients. A large-scale, randomized controlled study including males still needed to confirm the relationship between FSH and RA and disease activity.

In conclusion, high FSH seems to be a risk factor for RA and is positively associated with its disease activity. Despite the fact that the extragonadal effects of FSH have been studied in various menopausal symptoms, few studies have investigated the function and changes of serum FSH in women with RA. The mechanism of gender and age bias in autoimmune diseases is still not fully elucidated. Our findings provide new insight into the pathogenesis of RA. Inhibition of FSH levels may be a targeted therapy for RA.

## Data Availability Statement

The raw data supporting the conclusions of this article will be made available by the authors, without undue reservation.

## Ethics Statement

The studies involving human participants were reviewed and approved by the ethical committee of Shanxi Bethune Hospital. The patients/participants provided their written informed consent to participate in this study.

## Author Contributions

XZ, PQ, QG, FW, and XW contributed to the conception and design, and the initial draft of the manuscript. ZL and JP contributed to data analysis and revision. LZ contributed to supervision and editing. All authors contributed to the article and approved the submitted version.

## Funding

This work was supported by the Youth Foundation of Science and Technology Department of Shanxi Province (20210302123448) and the National Natural Science Foundation of China (81771768).

## Conflict of Interest

The authors declare that the research was conducted in the absence of any commercial or financial relationships that could be construed as a potential conflict of interest.

## Publisher’s Note

All claims expressed in this article are solely those of the authors and do not necessarily represent those of their affiliated organizations, or those of the publisher, the editors and the reviewers. Any product that may be evaluated in this article, or claim that may be made by its manufacturer, is not guaranteed or endorsed by the publisher.

## References

[1] Smolen JS Aletaha D McInnes IB. Rheumatoid Arthritis. Lancet (Lond Engl) (2016) 388(10055):2023–38. doi: 10.1016/S0140-6736(16)30173-8 27156434

[2] Favalli EG Biggioggero M Crotti C Becciolini A Raimondo MG Meroni PL. Sex and Management of Rheumatoid Arthritis. Clin Rev Allergy Immunol (2019) 56(3):333–45. doi: 10.1007/s12016-018-8672-5 29372537

[3] Pikwer M Bergström U Nilsson J Jacobsson L Turesson C. Early Menopause Is an Independent Predictor of Rheumatoid Arthritis. Ann Rheum Dis (2012) 71(3):378–81. doi: 10.1136/ard.2011.200059 21972241

[4] Weitzmann MN Pacifici R. Estrogen Deficiency and Bone Loss: An Inflammatory Tale. J Clin Invest (2006) 116(5):1186–94. doi: 10.1172/JCI28550 PMC1451218 16670759

[5] Abu-Taha M Rius C Hermenegildo C Noguera I Cerda-Nicolas J-M Issekutz AC. Menopause and Ovariectomy Cause a Low Grade of Systemic Inflammation That may be Prevented by Chronic Treatment With Low Doses of Estrogen or Losartan. J Immunol (Baltimore Md: 1950) (2009) 183(2):1393–402. doi: 10.4049/jimmunol.0803157 19553526

[6] Straub RH. The Complex Role of Estrogens in Inflammation. Endocr Rev (2007) 28(5):521–74. doi: 10.1210/er.2007-0001 17640948

[7] Reduction in Incidence of Rheumatoid Arthritis Associated With Oral Contraceptives. Royal College of General Practitioners’ Oral Contraception Study. Lancet (Lond Engl) (1978) 1(8064):569–71. 76118

[8] Vandenbroucke JP Valkenburg HA Boersma JW Cats A Festen JJ Huber-Bruning O. Oral Contraceptives and Rheumatoid Arthritis: Further Evidence for a Preventive Effect. Lancet (Lond Engl) (1982) 2(8303):839–42. doi: 10.1016/S0140-6736(82)90809-1 6126710

[9] Spector TD Brennan P Harris P Studd JW Silman AJ. Does Estrogen Replacement Therapy Protect Against Rheumatoid Arthritis? J Rheumatol (1991) 18(10):1473–6. 1765970

[10] Doran MF Crowson CS O’Fallon WM Gabriel SE. The Effect of Oral Contraceptives and Estrogen Replacement Therapy on the Risk of Rheumatoid Arthritis: A Population Based Study. J Rheumatol (2004) 31(2):207–13. 14760786

[11] Qi S Xin R Guo W Liu Y. Meta-Analysis of Oral Contraceptives and Rheumatoid Arthritis Risk in Women. Ther Clin Risk Manage (2014) 10:915–23 doi: 10.2147/TCRM.S70867. PMC4226450 25395857

[12] Wadström H Pettersson A Smedby KE Askling J. Risk of Breast Cancer Before and After Rheumatoid Arthritis, and the Impact of Hormonal Factors. Ann Rheum Dis (2020) 79(5):581–6. doi: 10.1136/annrheumdis-2019-216756 PMC7213316 32161056

[13] Taneja C Gera S Kim SM Iqbal J Yuen T Zaidi M. FSH-Metabolic Circuitry and Menopause. J Mol Endocrinol (2019) 63(3):R73–80. doi: 10.1530/JME-19-0152 PMC6992500 31454787

[14] Iqbal J Sun L Kumar TR Blair HC Zaidi M. Follicle-Stimulating Hormone Stimulates TNF Production From Immune Cells to Enhance Osteoblast and Osteoclast Formation. Proc Natl Acad Sci USA (2006) 103(40):14925–30. doi: 10.1073/pnas.0606805103 PMC1595452 17003115

[15] Qian H Jia J Yang Y Bian Z Ji Y. A Follicle-Stimulating Hormone Exacerbates the Progression of Periapical Inflammation Through Modulating the Cytokine Release in Periodontal Tissue. Inflammation (2020) 43(4):1572–85. doi: 10.1007/s10753-020-01234-9 32303868

[16] Doran MF Pond GR Crowson CS O’Fallon WM Gabriel SE. Trends in Incidence and Mortality in Rheumatoid Arthritis in Rochester, Minnesota, Over a Forty-Year Period. Arthritis Rheum (2002) 46(3):625–31. doi: 10.1002/art.509 11920397

[17] Davizon-Castillo P McMahon B Aguila S Bark D Ashworth K Allawzi A. TNF-α-Driven Inflammation and Mitochondrial Dysfunction Define the Platelet Hyperreactivity of Aging. Blood (2019) 134(9):727–40. doi: 10.1182/blood.2019000200 PMC6716075 31311815

[18] Zhang X-H Zhang Y-A Chen X Qiao P-Y Zhang L-Y. Assessment of the Ovarian Reserve by Serum Anti-Müllerian Hormone in Rheumatoid Arthritis Patients: A Systematic Review and Meta-Analysis. Int Arch Allergy Immunol (2021) 183(4):462–9. doi: 10.1159/000520133 34929705

[19] Pikwer M Giwercman A Bergstrom U Nilsson JA Jacobsson LT Turesson C. Association Between Testosterone Levels and Risk of Future Rheumatoid Arthritis in Men: A Population-Based Case-Control Study. Ann Rheum Dis (2014) 73(3):573–9. doi: 10.1136/annrheumdis-2012-202781 23553100

[20] Kass AS Lea TE Torjesen PA Gulseth HC Forre ØT. The Association of Luteinizing Hormone and Follicle-Stimulating Hormone With Cytokines and Markers of Disease Activity in Rheumatoid Arthritis: A Case-Control Study. Scandinavian J Rheumatol (2010) 39(2):109–17. doi: 10.3109/03009740903270607 20337546

[21] Cevik R Em S Gur A Nas K Sarac AJ Colpan L. Sex and Thyroid Hormone Status in Women With Rheumatoid Arthritis: Are There Any Effects of Menopausal State and Disease Activity on These Hormones? Int J Clin Pract (2004) 58(4):327–32. doi: 10.1111/j.1368-5031.2004.00005.x 15161114

[22] Baillargeon J Al Snih S Raji MA Urban RJ Sharma G Sheffield-Moore M. Hypogonadism and the Risk of Rheumatic Autoimmune Disease. Clin Rheumatol (2016) 35(12):2983–7. doi: 10.1007/s10067-016-3330-x PMC5544431 27325124

[23] Spector TD Perry LA Tubb G Silman AJ Huskisson EC. Low Free Testosterone Levels in Rheumatoid Arthritis. Ann Rheum Dis (1988) 47(1):65–8. doi: 10.1136/ard.47.1.65 PMC1003446 3345107

[24] Yang DD Krasnova A Nead KT Choueiri TK Hu JC Hoffman KE. Androgen Deprivation Therapy and Risk of Rheumatoid Arthritis in Patients With Localized Prostate Cancer. Ann Oncol: Off J Eur Soc Med Oncol (2018) 29(2):386–91. doi: 10.1093/annonc/mdx744 29267861

[25] Pope JE Joneja M Hong P. Anti-Androgen Treatment of Prostatic Carcinoma May Be a Risk Factor for Development of Rheumatoid Arthritis. J Rheumatol (2002) 29(11):2459–62. 12415609

[26] Cutolo M Balleari E Giusti M Monachesi M Accardo S. Sex Hormone Status in Women Suffering From Rheumatoid Arthritis. J Rheumatol (1986) 13(6):1019–23. 3104587

[27] Zhang C Zhao M Li Z Song Y. Follicle-Stimulating Hormone Positively Associates With Metabolic Factors in Perimenopausal Women. Int J Endocrinol (2020) 2020:7024321. doi: 10.1155/2020/7024321 33273916 PMC7676929

[28] Li Q Zheng D Lin H Zhong F Liu J Wu Y. High Circulating Follicle-Stimulating Hormone Level Is a Potential Risk Factor for Renal Dysfunction in Post-Menopausal Women. Front Endocrinol (Lausanne) (2021) 12:627903. doi: 10.3389/fendo.2021.627903 33868168 PMC8047631

[29] Ji Y Liu P Yuen T Haider S He J Romero R. Epitope-Specific Monoclonal Antibodies to Fshβ Increase Bone Mass. Proc Natl Acad Sci USA (2018) 115(9):2192–7. doi: 10.1073/pnas.1718144115 PMC5834707 29440419

[30] Xiong J Kang SS Wang Z Liu X Kuo T-C Korkmaz F. FSH Blockade Improves Cognition in Mice With Alzheimer’s Disease. Nature (2022) 603(7901):470–6. doi: 10.1038/s41586-022-04463-0 PMC9940301 35236988

[31] Islander U Jochems C Lagerquist MK Forsblad-d’Elia H Carlsten H. Estrogens in Rheumatoid Arthritis; the Immune System and Bone. Mol Cell Endocrinol (2011) 335(1):14–29. doi: 10.1016/j.mce.2010.05.018 20685609

[32] Kyaw MT Sakthiswary R Ani Amelia Z Rahana AR Munirah MM. Effects of Methotrexate Therapy on the Levels of Gonadotropic Hormones in Rheumatoid Arthritis Patients of Reproductive Age. Cureus (2020) 12(4):e7632. doi: 10.7759/cureus.7632 32399364 PMC7213647

[33] Wong LE Huang WT Pope JE Haraoui B Boire G Thorne JC. Effect of Age at Menopause on Disease Presentation in Early Rheumatoid Arthritis: Results From the Canadian Early Arthritis Cohort. Arthritis Care Res (Hoboken) (2015) 67(5):616–23. doi: 10.1002/acr.22494 25303739

