# Corrigendum: High follicle-stimulating hormone level associated with risk of rheumatoid arthritis and disease activity

**DOI:** 10.3389/fendo.2023.1238584

**Published:** 2023-06-23

**Authors:** Xianhui Zhang, Pengyan Qiao, Qianyu Guo, Zixie Liang, Jie Pan, Fengping Wu, Xuexue Wang, Liyun Zhang

**Affiliations:** ^1^ Department of Rheumatology, Third Hospital of Shanxi Medical University, Shanxi Bethune Hospital, Shanxi Academy of Medical Sciences, Tongji Shanxi Hospital, Taiyuan, China; ^2^ Department of Biomedical Engineering, Duke University Pratt School of Engineering, Durham, NC, United States; ^3^ Department of Pathology, Stanford University School of Medicine, Palo Alto, CA, United States; ^4^ School of Basic Medical Sciences, Shanxi Medical University, Taiyuan, China

**Keywords:** FSH, menopause, rheumatoid arthritis, disease activity, sex hormones

In the published article, there was an error in affiliation 1. Instead of “Department of Rheumatology, Third Hospital of Shanxi Medical University, Shanxi Bethune Hospital, Shanxi Academy of Medical Sciences, Tongji Shanxi Hospital, Tongji Medical College, Huazhong University of Science and Technology, Taiyuan, China”, it should be “Department of Rheumatology, Third Hospital of Shanxi Medical University, Shanxi Bethune Hospital, Shanxi Academy of Medical Sciences, Tongji Shanxi Hospital, Taiyuan, China”.

In the published article, there was also an error regarding the affiliation for Liyun Zhang. Instead of having affiliation 5 *Department of Rheumatology, Shanxi Bethune Hospital, Shanxi Academy of Medical Sciences; Tongji Shanxi Hospital, Tongji Medical College, Huazhong University of Science and Technology, Taiyuan, China*, they should have affiliation 1 *Department of Rheumatology, Third Hospital of Shanxi Medical University, Shanxi Bethune Hospital, Shanxi Academy of Medical Sciences, Tongji Shanxi Hospital, Taiyuan, China*. Affiliation 5 has now been removed.

The authors apologize for these errors and state that this does not change the scientific conclusions of the article in any way. The original article has been updated.

